# Glycaemic control boosts glucosylated nanocarrier crossing the BBB into the brain

**DOI:** 10.1038/s41467-017-00952-3

**Published:** 2017-10-17

**Authors:** Y. Anraku, H. Kuwahara, Y. Fukusato, A. Mizoguchi, T. Ishii, K. Nitta, Y. Matsumoto, K. Toh, K. Miyata, S. Uchida, K. Nishina, K. Osada, K. Itaka, N. Nishiyama, H. Mizusawa, T. Yamasoba, T. Yokota, K. Kataoka

**Affiliations:** 1 Department of Bioengineering, Graduate School of Engineering, The University of Tokyo, 7-3-1 Hongo, Bunkyo-ku, Tokyo 113-8656 Japan; 2 Department of Neurology and Neurological Science, Graduate School of Medical and Dental Sciences, Tokyo Medical and Dental University (TMDU), 1-5-45 Yushima, Bunkyo-ku, Tokyo 113-8519 Japan; 3 Center for Brain Integration Research, Tokyo Medical and Dental University (TMDU), 1-5-45 Yushima, Bunkyo-ku, Tokyo 113-8519 Japan; 4 Center for Disease Biology and Integrative Medicine, Graduate School of Medicine, The University of Tokyo, 7-3-1 Hongo, Bunkyo-ku, Tokyo 113-0033 Japan; 5 Department of Otorhinolaryngology and Head and Neck Surgery, Graduate School of Medicine and Faculty of Medicine, The University of Tokyo, 7-3-1 Hongo, Bunkyo-ku, Tokyo 113-0033 Japan; 6 Innovation Center of NanoMedicine, Kawasaki Institute of Industrial Promotion, 3-25-14 Tonomachi, Kawasaki-ku, Kawasaki 210-0821 Japan; 7 Department of Materials Engineering, Graduate School of Engineering, The University of Tokyo, 7-3-1 Hongo, Bunkyo-ku, Tokyo 113-8656 Japan; 8 Laboratory for Chemistry and Life Science, Institute of Innovative Research, Tokyo Institute of Technology, R1-11, 4259 Nagatsuta, Midori-ku, Yokohama 226-8503 Japan; 9 Policy Alternatives Research Institute, The University of Tokyo, 7-3-1 Hongo, Bunkyo-ku, Tokyo 113-0033 Japan

## Abstract

Recently, nanocarriers that transport bioactive substances to a target site in the body have attracted considerable attention and undergone rapid progression in terms of the state of the art. However, few nanocarriers can enter the brain via a systemic route through the blood-brain barrier (BBB) to efficiently reach neurons. Here we prepare a self-assembled supramolecular nanocarrier with a surface featuring properly configured glucose. The BBB crossing and brain accumulation of this nanocarrier are boosted by the rapid glycaemic increase after fasting and by the putative phenomenon of the highly expressed glucose transporter-1 (GLUT1) in brain capillary endothelial cells migrating from the luminal to the abluminal plasma membrane. The precisely controlled glucose density on the surface of the nanocarrier enables the regulation of its distribution within the brain, and thus is successfully optimized to increase the number of nanocarriers accumulating in neurons.

## Abstract (web summary)

There are only a few examples of nanocarriers that can transport bioactive substances across the blood-brain barrier. Here the authors show that by rapid glycaemic increase the accumulation of a glucosylated nanocarrier in the brain can be controlled.

## Introduction

Recent advances in nanotechnology have allowed the development of nanocarriers based on the controlled self-assembly of precisely designed molecules as drug vehicles with various different shapes and sizes^1–7^. These nanocarriers are a promising system for delivering therapeutic or diagnostic agents to diseased sites in the body, particularly to solid tumours with highly permeable blood vessels^8–10^. In fact, clinical trials of nanocarriers for tumour-targeted delivery are currently in progress^11–14^. Nevertheless, designing nanocarriers that transport biologically active substances through the barrier of poorly permeable blood vessels remains a challenging task. In particular, the brain is strongly protected by the blood–brain barrier (BBB), a system consisting primarily of brain capillary endothelial cells (BCECs) along with pericytes and astrocytes^15, 16^. Only small hydrophobic molecules with a molecular weight lower than ~450 can penetrate the BBB^15^. Because the intractability of many central nervous system (CNS) disorders arises from the BBB inhibiting the delivery of various drugs into the brain, developing a method of using nanocarriers to efficiently deliver drugs through the BBB is of the highest priority. Many efforts towards transporting nanocarriers into the brain have been reported to date. A recent example is a BBB-crossing nanocarrier equipped with peptides capable of recognizing the transferrin receptor on BCECs; however, its accumulation rate in the brain did not exceed 1.0% dose/g-brain^17^. In another example, apolipoprotein E-bound nanoparticles crossed the BBB to reach neurons, but the delivery efficiency was not reported^18^. Among the various candidate ligands previously studied for promoting BBB traversal, glucose, the main energy source in the brain, is notable because glucose transporter-1 (GLUT1) is expressed at a remarkably high level compared to many other receptors and transporters in BCECs^19^. Although several nanocarriers targeting GLUT1 by incorporating glucose as the ligand have been designed^20, 21^, none of them have been transported into the brain at a high level. Therefore, the GLUT1-based delivery strategy requires an innovative approach.

Here we report a biological strategy that exploits the rapid glycaemic increase after fasting to enable a remarkably enhanced delivery of a nanocarrier across the BBB via GLUT1.

## Results

### Preparation of glucose-integrated nanocarriers

**Fig. 1:**
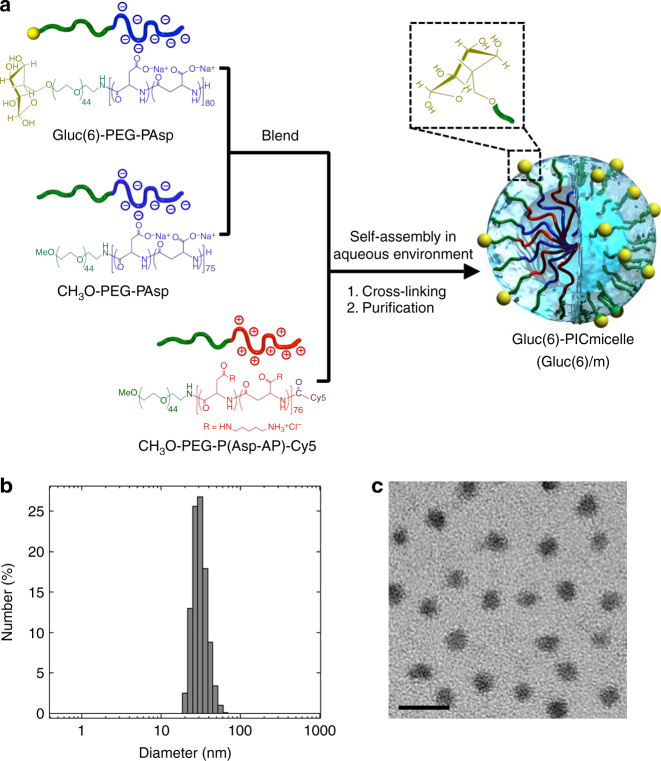
Characterization of the Gluc(6)-conjugated PIC micelle (Gluc(6)/m). **a** Scheme of Gluc(6)/m preparation via the assembly of oppositely charged block copolymers. **b** Size distribution of the 25%Gluc(6)/m determined by DLS. **c** TEM image of the 25%Gluc(6)/m. The scale bar indicates 50 nm

GLUT1 recognizes glucose molecules principally through weak interactions such as hydrogen bonding and the hydrophobic effect (*K*_m_ = 1.5 mM)^22^. Therefore, the binding of a single glucose to GLUT1 may not be strong enough to retain nanocarriers in the harsh flow of the bloodstream. A promising strategy to achieve strong retention of nanocarriers on BCECs through GLUT1 binding is to induce a multivalent interaction between a nanocarrier and multiple GLUT1 molecules on BCECs (polyvalent effect^23, 24^) by introducing many glucose molecules onto the surface of the nanocarrier that is sufficiently larger in size than a single GLUT1 molecule (~3 nm)^25^. For this purpose, we constructed a polymeric micellar nanocarrier with a size of ~30 nm via the multimolecular association of oppositely charged pairs of polyethylene glycol (PEG)-based block ionomers. The surface of the polymeric micelles was decorated with multiple glucose molecules with a controlled density (Fig. 1a). Glucose was introduced onto the micelles via an ether linkage at the C6 position to preserve the binding ability to GLUT1 because the OH groups at positions C1, C3 and C4 of glucose are believed to be essential for its interaction with GLUT1^26^. Specifically, a PEG-poly(*α*,*β*-aspartic acid) block copolymer (PEG-PAsp) with glucose conjugated to the *α*-end of the PEG segment via a bond with the C6-O moiety (Gluc(6)-PEG-PAsp) (*M*_n_ of PEG = 2000, DP of P(Asp) = 80) was blended in an aqueous solution at an arbitrary proportion with CH_3_O-PEG-PAsp (*M*_n_ of PEG = 2000, DP of P(Asp) = 75) (blending ratio of Gluc(6)-PEG-PAsp to CH_3_O-PEG-PAsp = 1:0, 1:1, 1:4 and 0:1). This solution was then mixed with an aqueous solution of oppositely charged CH_3_O-PEG-poly([5-aminopentyl]-*α,β*-aspartamide) block copolymer (*M*_n_ of PEG = 2000, DP of P(Asp-AP) = 76) labelled with a fluorescence dye (Cy5) at the *ω*-end (CH_3_O-PEG-P(Asp-AP)-Cy5) at a charge ratio of 1:1 and stabilized by crosslinking with 1-ethyl-3-(3-dimethylaminopropyl) carbodiimide. In this manner, a series of polyion complex (PIC) micelles consisting of a crosslinked PIC core covered by a hydrophilic, non-charged PEG shell with a constant size and systemically altered surface glucose densities (Gluc(6)-conjugated PIC micelles (X%Gluc(6)/m, *X* = 10, 25 and 50)) were prepared (Fig. 1a). For the control micelles that may not bind to GLUT1, PIC micelles without the surface glucose (Null/m) and those with glucose conjugated via an ether linkage at the C3 position (Gluc(3)/m) were prepared. Dynamic light scattering (DLS) measurement and transmission electron microscopy (TEM) observation revealed that the PIC micelles are monodisperse (polydispersity index <0.1) with a diameter of 30 nm regardless of the glucose density (0, 10, 25 or 50%) or the conjugating position of glucose (Gluc(6) or Gluc(3)) (Fig. 1b, c, Supplementary Fig. 12 and Supplementary Table 1).

Next, we assessed the binding property of the PIC micelles to GLUT1 in vitro. For this purpose, we generated a GLUT1-overexpressing stable cell line by transfecting mouse GLUT1-expressing plasmid (Supplementary Fig. 13A, B), added the micelles with or without the surface glucose to the medium of these cells on a culture plate and quantified their uptake into the cells by measuring the fluorescent intensities of the detached cell suspension. The uptake into GLUT1-overexpressing cells, not into mock-transfected cells, was increased for the Gluc(6)/m (Supplementary Fig. 13C), and this increase of uptake was completely inhibited by the standard GLUT1 inhibitors cytochalasin B and phloretin, which have different mechanisms of action (Supplementary Fig. 13D). The cellular uptake of the Gluc(3)/m was comparable to that of the Null/m, excluding the possibility of a nonspecific sugar–mucosal interaction (Supplementary Fig. 13C). These data indicate that the glucose molecules attached via the C6 position onto the micelle surface indeed can bind to mouse GLUT1 expressed on the cell membrane.

### Glycaemic control boosts nanocarrier accumulation in the brain

**Fig. 2:**
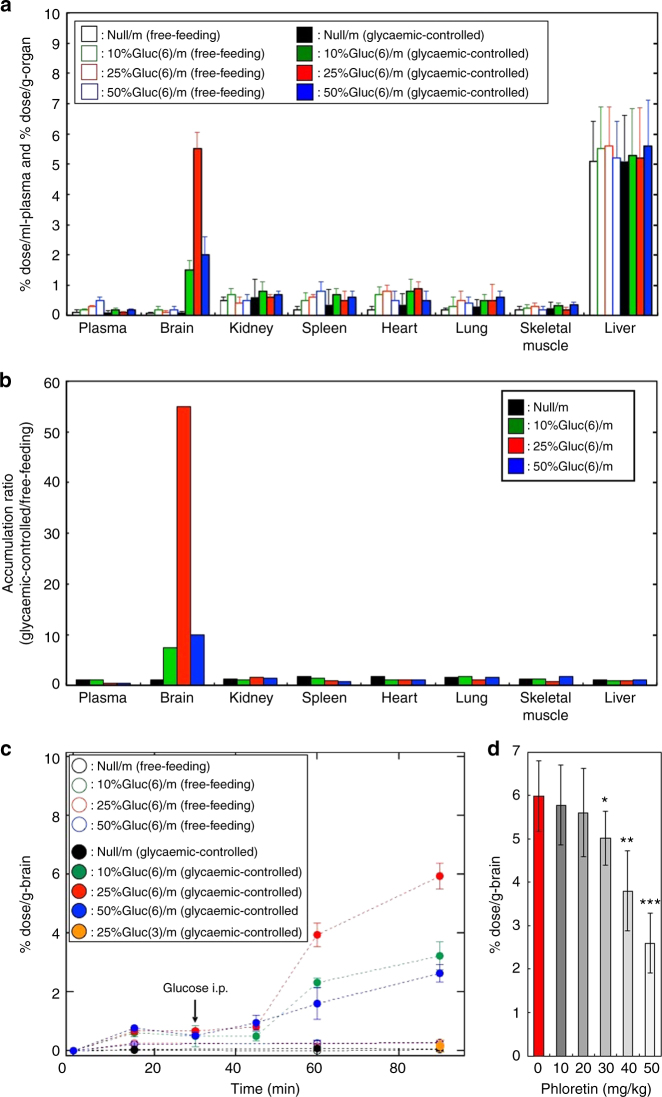
Pharmacokinetic profiles of the polymeric micelles. **a** Biodistribution of micelles (Null/m and Gluc(6)/m) in mice under different feeding conditions at 48 h after the injection. Open and closed bars show free-feeding and glycaemic-controlled groups, respectively. Black, green, red and blue coloured bars indicate Null/m, 10%Gluc(6)/m, 25%Gluc(6)/m and 50%Gluc(6)/m, respectively. **b** Accumulation ratio in mice (glycaemic-controlled/free-feeding) of each micelle at 48 h calculated from the values shown in **a**. **c** Time course of micelle accumulation (Null/m, Gluc(6)/m and Gluc(3)/m) in the mouse brain under different feeding conditions. The arrow in **c** indicates when 20 wt% glucose was intraperitoneally injected. Open and closed circles denote the free-feeding and glycaemic-controlled groups, respectively. Black, green, red and blue coloured circles indicate Null/m, 10%Gluc(6)/m, 25%Gluc(6)/m and 50%Gluc(6)/m, respectively. An orange coloured circle shows 25%Gluc(3)/m (glycaemic-controlled). **d** In vivo inhibition study of the brain accumulation of the 25%Gluc(6)/m by phloretin. Red and grey bars show experiments conducted without and with phloretin, respectively. Experiments were performed using BALB/c mice (female, 6-week-old, *n* = 5). The data are expressed as the mean ± SEM for **a**, **c**, **d** and as the mean for **b**. *P < 0.05

We next examined the pharmacokinetic profiles of our polymeric micelles upon intravenous administration into normal free-feeding mice. All micelles irrespective of the presence of surface glucose showed prolonged retention in blood: more than 80% of the initial dose remained in the blood circulation 90 min after an intravenous injection (Supplementary Fig. 14). At 48 h after injection, the accumulation of the micelles in most organs, including the brain, was very limited to below 1% dose/g-organ, even for the Gluc(6)/m (Fig. 2a, open bars). The accumulation in the liver was up to 5–6% dose/g-liver, and the accumulation was not increased by decorating with glucose (Fig. 2a, open bars), suggesting that the liver uptake of the micelles was mostly by a nonspecific mechanism. These results indicate that the strategy of only decorating the nanocarriers with glucose does not have a substantial effect on the delivery of polymeric micelles into the brain.

We then considered introducing glycaemic control with the animal model. Lowering the blood glucose concentration was reported to facilitate GLUT1 localization on the luminal plasma membrane in BCECs^27^; therefore, GLUT1 would be internalized into BCECs based on an elevation of blood glucose concentration. Based on this assumption, we investigated the effect of glycaemic control on the accumulation of our micelles in the brain by intravenously injecting the micelles into mice after a 1-day fast and intraperitoneally administering a 20 wt% glucose solution 30 min after the micelle injection to elevate the blood glucose concentration. The accumulation of the micelles in most organs regardless of the presence of the surface glucose was unchanged by introducing this glycaemic control. However, a remarkable and specific increase in the micelle accumulation in the brain was observed in only the Gluc(6)/m-administered mice (Fig. 2a, b, closed bars). The surface glucose density of the Gluc(6)/m had substantial effect on accumulation, with the 25%Gluc(6)/m exhibiting the highest accumulation rate of up to 6% dose/g-brain (Fig. 2a, red closed bar), which was 56 times higher than the rate observed in free-feeding mice (Fig. 2b, red closed bar). An accumulation rate of 6% dose/g-brain is appealing because it is significantly higher than those reported to date for other carrier systems using glucose or peptide ligands^17, 20, 21^ and is almost equal to the enhanced permeability and retention effect (EPR)-driven accumulation rate of long-circulating stealth nanocarriers into tumours with highly leaky vasculatures^1, 8, 9^.

An evaluation of the time course revealed that when the 20 wt% glucose solution was administered to fasting mice subsequent to the micelle injection, a remarkable increase in the micelle accumulation into the brain starting ~15 min after the glucose administration was observed only in the Gluc(6)/m-administered mice (Fig. 2c). Importantly, Gluc(3)/m failed to achieve a similarly remarkable accumulation in the brain (the accumulation rate was comparable to that of Null/m (0.2%)) after the same glycaemic control (Fig. 2c). This notable difference in the brain accumulation rate between Gluc(6)/m and Gluc(3)/m strongly suggests the involvement of GLUT1 because the C1, C3 and C4-OH groups in glucose are essential for the recognition by GLUT1^26^. As C3-OH in Gluc(3)/m was substituted with an ether linkage to PEG, Gluc(3)/m is assumed to have low affinity towards GLUT1. Moreover, the involvement of GLUT1 was suggested by the considerable decrease in the accumulation of 25%Gluc(6)/m in the brain (Fig. 2d) upon intravenous administration of phloretin (a GLUT1 inhibitor).

Although there was a previous study on the brain delivery of glucose-decorated PEGylated liposomes, no glycaemic manipulation of fasting animals was conducted and the accumulation rate was limited to only 0.4% dose/g-brain^20^. This result is consistent with our results obtained for the mice that were not intraperitoneally administered glucose (~0.3% dose/g-brain), suggesting that the manipulation of blood glucose concentration is essential for achieving significant and selective accumulation of glucose-integrated nanocarriers into the brain.

### Direct observation of nanocarriers crossing the BBB

**Fig. 3:**
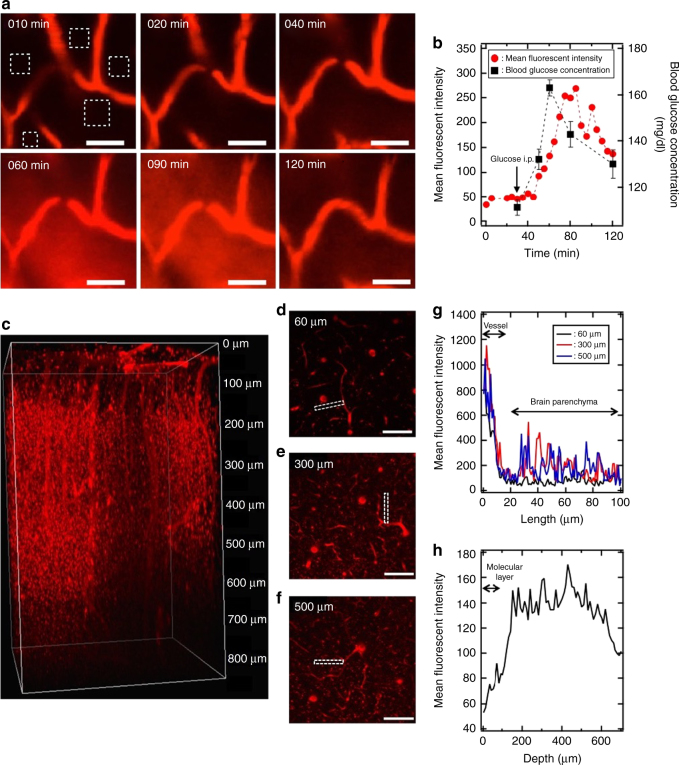
Real-time observation of the 25%Gluc(6)/m crossing the BBB. **a** Sequential images of mouse cerebrum observed using IVRT-CLSM. The 25%Gluc(6)/m (red) were intravenously injected to a mouse after a 24-h fast, followed by an intraperitoneal injection of 20 wt% glucose 30 min later. The scale bars indicate 50 μm. **b** Time course of blood glucose concentration (black squares) and the mean fluorescent intensities of the 25%Gluc(6)/m (red circles) in the region of interest (ROI) of the brain parenchyma indicated as white rectangles in **a**. The arrow indicates the time of intraperitoneal injection of the 20 wt% glucose. Blood glucose concentrations were expressed as the mean ± SEM. **c** Images of Gluc(6)/m (red) in the mouse cerebrum observed using intravital multiphoton microscopy 48 h after administration. Cross-sections at depths of 60 μm (**d**), 300 μm (**e**) and 500 μm (**f**) are also shown. The scale bars indicate 100 μm. **g** Distribution profiles of Gluc(6)/m from blood vessels to the brain parenchyma in the selected region (indicated by a white rectangle in **d**, **e**, **f**) at depths of 60 μm (black line), 300 μm (red line) and 500 μm (blue line). **h** Depth profiles of the mean fluorescent intensities from 0 to 700 μm in the brain parenchymal region

After establishing the outstanding accumulation of 25%Gluc(6)/m in the brain, we attempted to directly observe the micelles crossing the BBB using intravital real-time confocal laser-scanning microscopy (IVRT-CLSM). IVRT-CLSM allows spatiotemporal and quantitative analysis of behaviours such as the extravasation and tissue crossing of nanocarriers in a living animal^28^. 25%Gluc(6)/m was injected intravenously into fasting mice, followed by an administration of the 20 wt% glucose solution 30 min later. In this experiment, we evaluated the real-time extravasation of the micelles through the BBB based on the intensity and distribution of fluorescent signals. Figure 3a shows IVRT-CLSM micrographs of the cerebrum. Initially, at 10 min after the administration of the 25%Gluc(6)/m (before the administration of the glucose solution), fluorescent signals (red) were observed only inside of the vasculature. Next, at 60 min after the administration of the 25%Gluc(6)/m (30 min after the administration of the glucose solution), the fluorescent signals were observed extensively throughout the brain parenchyma (the areas apart from the bloodstream) and then further increased in intensity until 90 min. To quantitatively evaluate the micelles crossing the BBB, five different regions in the brain parenchyma were selected (squares enclosed by white dotted lines in Fig. 3a), and the fluorescent intensities were measured until 120 min (Fig. 3b, red circles). The blood glucose concentration was monitored at 30, 50, 60, 80 and 120 min after administering the 25%Gluc(6)/m (Fig. 3b, black squares). The fluorescent intensities in the brain parenchyma started to increase at ~10–20 min after the injection of glucose solution, i.e., as the blood glucose concentration increased. The decay of the fluorescent intensities after the peak also closely followed the decrease in the blood glucose concentration.

Notably, at 48 h after the micelle administration, intravital multiphoton microscopy clearly showed the fluorescent signals of the 25%Gluc(6)/m in the extravascular parenchyma of the cerebrum at a depth of up to 700 μm from the cortical surface (Fig. 3c and Supplementary Movie 1). The cross-section images showed only slight 25%Gluc(6)/m distribution in the molecular layer where cellular components were scarce (Fig. 3d, g, h); by contrast, marked 25%Gluc(6)/m distribution was observed in the deeper layers where cellular components were abundant (Fig. 3e–h), suggesting that the micelles were taken up by parenchymal cells.

### Distribution profiles of nanocarriers in the brain parenchyma

**Fig. 4:**
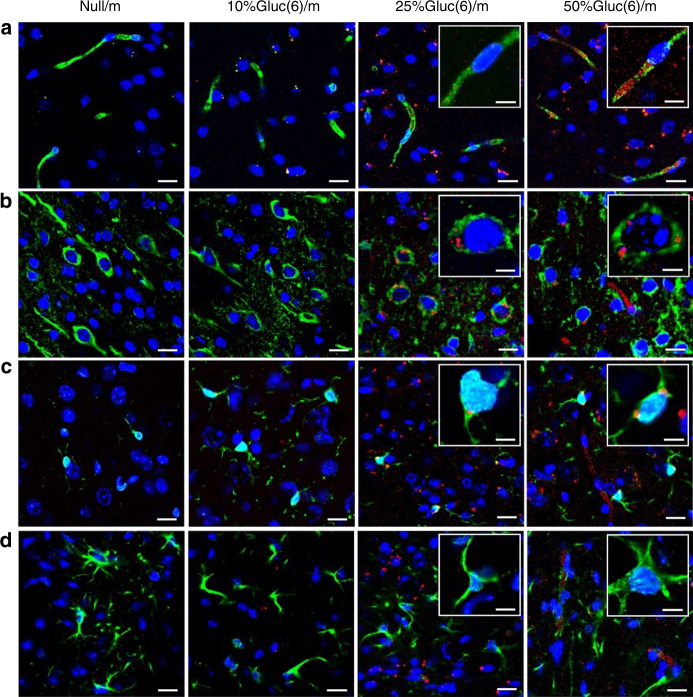
Immunohistochemical analysis of the mouse brains after administration of polymeric micelles. Cerebral sections at 48 h after the administration of Null/m, 10%Gluc(6)/m, 25%Gluc(6)/m and 50%Gluc(6)/m (red). BCECs (**a**), neurons (**b**), microglia (**c**) and astrocytes (**d**) (green) are stained with anti-PECAM1, anti-Tuj1, anti-Iba1 and anti-GFAP antibodies, respectively. Nuclei (blue) are stained with DAPI. The scale bar indicates 20 μm (10 μm in insets)

We then conducted immunohistochemical analysis of brain specimens to determine the destination of the micelles. For this purpose, we first intravenously administered Null/m, 10%Gluc(6)/m, 25%Gluc(6)/m or 50%Gluc(6)/m to fasting mice and then intraperitoneally injected them with 20 wt% glucose 30 min later. We obtained the mice brains 48 h after the administration of the micelles and stained the frozen sections with antibodies against PECAM1, Tuj1, Iba1 and GFAP to detect BCECs, neurons, microglia and astrocytes, respectively. The fluorescent signals of the micelles in the brain sections were then observed using confocal laser-scanning microscopy (Fig. 4). The Null/m were not recognized in any region of the brain. Although the 50%Gluc(6)/m were clearly distributed throughout the BCECs, the 10%Gluc(6)/m and 25%Gluc(6)/m were barely noticeable in this region (Fig. 4a), suggesting that a considerable fraction of the 50%Gluc(6)/m stayed in and/or around the BCECs. An appreciable fraction of the 25%Gluc(6)/m and 50%Gluc(6)/m were incorporated into the neurons and microglia (Fig. 4b, c). As shown in Fig. 4d, we did not identify the accumulation of any micelles in the astrocytes regardless of the glucose density. The relatively higher accumulation of 50%Gluc(6)/m in the BCECs can be explained as a manifestation of their lower dissociation constant (*K*_d_) to GLUT1 due to the high surface glucose density, which leads to the stacking of the Gluc(6)/m in and/or around the BCECs. Importantly, further investigations are needed to clarify the precise delivery route of Gluc(6)/m after crossing the BCECs, especially from the perspective of the micellar distribution within the neurovascular unit, which covers a wider range of components in the CNS^29^.

### Delivery mechanism of the nanocarriers across the BCECs

Our study has revealed that the following three factors are critical for efficiently transporting glucose-integrated nanocarriers into the brain: (1) introducing multiple properly-oriented glucose molecules onto the surface of a polymeric micelle, (2) administering the micelle to fasting mice and (3) subsequently increasing blood glucose concentration.

In BCECs, GLUT1 is localized on both the luminal and abluminal plasma membranes, although the relative distribution is not uniform across species^30^. Additionally, GLUT1 is internalized via clathrin-independent endocytosis, and is translocated between intracellular compartments and the plasma membrane as a physiological phenomenon of intracellular recycling^31–33^, which is conceptually similar to the behaviour of glucose transporter-4 (GLUT4) in adipose and muscular cells in response to insulin^34, 35^. The luminal plasma membrane levels of GLUT1 protein increase by more than 50% under hypoglycaemic conditions when compared with the levels under normal and hyperglycaemic conditions because of a redistribution that favours luminal transporters and an increase in GLUT1 synthesis at the gene expression level^27^. This phenomenon suggests that GLUT1 migrates from the cellular interior onto the plasma membrane surface of BCECs depending on the demand for glucose in the brain. Although there is no published evidence for GLUT1 translocation between the luminal and abluminal membrane in BCECs, the above characteristics of intracellular GLUT1 migration led us to the following interpretation: after attaching to GLUT1 on the luminal plasma membrane of BCECs, our nanocarrier was partly transcytosed through the BCECs to an abluminal site during the intracellular recycling of GLUT1 in response to a glycaemic change.

We then conducted immunohistochemical studies on the brain sections to obtain insight into the mechanism of the Gluc(6)/m crossing the BCECs by visualizing their recycling endosomes. For this purpose, we intravenously injected fasting mice with 25%Gluc(6)/m or 50%Gluc(6)/m, then intraperitoneally administered them with glucose solution after 30 min and finally obtained the brains after an additional 30 min because we could estimate from the findings shown in Figs. 2 and 3 that the Gluc(6)/m were in the process of crossing the BCECs at this time point. Eventually, both 25%Gluc(6)/m and 50%Gluc(6)/m were partially localized at recycling endosomes immunolabelled with anti-Rab11a antibody (Supplementary Fig. 15A, B). When the glycaemic increase was not evoked, weak signals of the Gluc(6)/m were found within the BCECs, but no signals were localized in the recycling endosomes (Supplementary Fig. 15C, D). These observations are consistent with the proposed scheme that within BCECs, Gluc(6)/m may migrate together with the recycling endosomes in response to a glycaemic increase after a fasting state. Because the migration of GLUT4 within the intracellular recycling process takes ~12–35 min^35^, we assume that the delay of 10–30 min between the elevation of blood glucose concentration and the accumulation of the Gluc(6)/m in the brain is consistent with our hypothesis that the delivery mechanism involves the intracellular recycling of GLUT1.

The intracellular migration of GLUT1 in BCECs must be controlled by multiple factors, including an endocrinological response and signal transduction within the neural circuit in relation to the blood glucose concentration, to maintain energy homoeostasis in vivo. A detailed physiological study would be necessary to fully explain the glycaemic-dependent delivery mechanism of our glucosylated nanocarriers across the BBB.

## Discussion

We developed polymeric micelles decorated with properly configured glucose molecules to recognize GLUT1 on BCECs and successfully demonstrated their preferential and efficient crossing of the BBB induced by a glycaemic increase after a fasting state. The appeal of this system is the on–off switch delivery strategy, in which the transport of the ligand (glucose)-conjugated nanocarrier into the brain may be facilitated by an external trigger (glucose) inducing the intracellular recycling of transporter molecules (GLUT1). Indeed, the involvement of GLUT1 in the delivery of the glucose-conjugated polymeric micelles into the brain was verified experimentally, and the transport of the micelles through the BBB into the brain parenchyma was directly observed in situ under IVRT-CLSM. Furthermore, managing the glucose density on the polymeric micelle enabled cellular-level control over the destination in the brain: the 25%Gluc(6)/m are mostly directed to the neurons and microglia, whereas the 50%Gluc(6)/m primarily remain in and/or around the BCECs. These results suggest that the nanocarrier system reported here has potential for delivering various drugs directly into the brain by crossing the BBB. Nevertheless, in diseased conditions, GLUT1 in BCECs might have an altered expression level^36^ and/or might not similarly translocate from the luminal to abluminal plasma membrane. Additionally, in diseases such as glioma, the BBB integrity can be disrupted^37^. Thus, the clinical relevance of the present results should be further examined by using animal models that properly reflect the BBB structure in each disease and by optimizing the structure of the glucosylated nanocarriers and the condition of glycaemic control.

## Methods

### Materials and animals

Information regarding materials and animals is described in the “Methods” section of Supplementary Information. All animal experiments were performed in accordance with the Guidelines for the Care and Use of Laboratory Animals, as stated by the University of Tokyo and Tokyo Medical and Dental University.

### Polymer synthesis

Detailed procedures for the preparation and characterization of CH_3_O-PEG-PAsp, CH_3_O-PEG-P(Asp-AP)-Cy5, Gluc(6)-PEG-PAsp and Gluc(3)-PEG-PAsp are provided in the Supplementary Figs. 1–11.

### Preparation and characterization of polymeric micelles

Detailed preparation schemes of the Gluc(6)/m, Gluc(3)/m and Null/m are given in the Supplementary Fig. 12 and Supplementary Table 1. The size distribution of the polymeric micelles was evaluated by conducting DLS measurements at 25 °C in 10 mM phosphate buffer at pH 7.4 using a Zetasizer Nano ZS90 (Malvern Instruments Ltd., Worcestershire, UK). TEM images of the polymeric micelles were obtained with a JEM-1400 (JEOL Ltd., Tokyo, Japan) according to the following sample preparation procedure: mesh copper grids were coated with a thin film of collodion followed by carbon coating, and a 0.1 mg/ml sample solution was placed on these carbon-coated grids. The sample on the grid was stained with 2 wt% uranyl acetate in a mixture of water and ethanol (v/v = 1/1) and then blotted to remove any extra stain.

### Biodistribution study

To analyse the distribution of the Gluc(6)/m, Gluc(3)/m and Null/m in vivo, BALB/c mice (*n* = 4) were reared without food for 24 h. Then, these mice were intravenously injected with 200 μl of 1 mg/ml Cy5-labelled polymeric micelle (Gluc(6)/m, Gluc(3)/m or Null/m) in D-PBS(−) and, after 30 min, intraperitoneally injected with 200 μl of 20 wt% D-(+)-glucose solution in D-PBS(−). The mice were killed at 15, 30, 45, 60 and 90 min and 48 h after the glucose injection and the excess blood was washed out by perfusion with D-PBS(−). The brain, liver, kidneys, spleen, heart, lung and femoral muscle were excised, washed with D-PBS(−), weighed after removing excess fluid and homogenized with cell lysis buffer. Blood was collected from the inferior vena cava, heparinized and centrifuged to obtain plasma. The accumulated amount of the micelles was quantified by fluorescence measurement using an Infinite M1000 PRO spectrophotometer (Tecan Group Ltd., Männedorf, Switzerland).

### Effect of phloretin on brain accumulation of nanocarrier

To examine how the brain accumulation of the 25%Gluc(6)/m was effected by the inhibition of GLUT1, BALB/c mice (*n* = 4) were fasted for 24 h and then intravenously injected with the solution of the 25%Gluc(6)/m. After 30 min, they were intraperitoneally injected with 200 μl of 20 wt% glucose solution, followed by an intravenous injection of the GLUT1 inhibitor phloretin (10–50 mg/kg)^38^ at 20 min after glucose administration. The brain was extracted in the same manner as biodistribution study, and the accumulation of the 25%Gluc(6)/m in the brain was quantified by fluorescence measurement using the Infinite M1000 PRO (Tecan Group Ltd.).

### Intravital real-time confocal laser-scanning microscopy

The mice were anaesthetized with 2.5% isoflurane (Abbott, North Chicago, IL) using a Univentor 400 anaesthesia unit (Univentor; Zetjun, Malta). The skulls of mice were partially cut open around the ROIs without damaging blood vessels. The mice were directly placed on a thermoplate (Tokai Hit, Tokyo, Japan), and a cover slip (Muto Pure Chemicals, Tokyo, Japan) was attached with proper pressure to flatten the brain surface. Images of the micelle crossing the BBB were acquired with an A1R confocal laser-scanning microscopy system (Nikon Corp., Tokyo, Japan) attached to an upright ECLIPSE FN1 (Nikon Corp.).

### Intravital multiphoton microscopy

Depth profile images of the micelle brain distribution were collected on a multiphoton microscope system equipped with a pulse laser (InSight DeepSee, Spectra Physics, Santa Clara, CA) and an Apochromat ×25 water immersion objective lens (2.0 mm WD, 1.10 NA) (Nikon A1R MP, Nikon Corp.). Imaging was performed using an excitation wavelength tuned at 1100 nm. The mice were anaesthetized with 1.5–2% isoflurane, and the head was stabilized with a head holder (SGM-4, Narishige Co., Ltd., Tokyo, Japan). The scalp and dura were removed to create a 3-mm diameter cranial window over the right hemisphere (2 mm lateral from the midline, 2 mm posterior to the bregma). After removing the skull bone, a glass bottom dish (diameter 40 mm, thickness 0.17 mm, WillCo-dish^®^, WillCo Wells B.V., Amsterdam, the Netherlands) was attached to the exposed brain tissue.

### Immunohistochemical analysis of brain sections

The mice were deprived of food for 24 h and then intravenously injected with the solution of the Cy5-labelled polymeric micelles (Null/m, 10%Gluc(6)/m, 25%Gluc(6)/m and 50%Gluc(6)/m, respectively), followed by an intraperitoneal injection of 20 wt% glucose solution. After 48 h, the mice were killed and were perfused with PBS and 4% paraformaldehyde. The brains were fixed overnight in 4% paraformaldehyde at 4 °C and sequentially soaked overnight in 20 wt% sucrose solution at 4 °C. The fixed specimens were snap-frozen in liquid nitrogen and then were sliced into 14-μm-thick sections using a CM3050 S cryostat (Leica Microsystems, Wetzler, Germany). The sections were stained with DAPI to visualize the nuclei and immunolabelled with antibodies against PECAM1 (1:200), Tuj1 (1:100), Iba1 (1:300) and GFAP (1:200) to visualize BCECs, neurons, microglia and astrocytes, respectively. Next, the sections were incubated with an Alexa Fluor 488-conjugated secondary antibody. All images were acquired using the A1R confocal laser-scanning microscope (Nikon Corp.).

### Data availability

The data that support the findings reported herein are available on reasonable request from the corresponding authors.

## Electronic supplementary material

Supplementary Information

Description of Additional Supplementary Files

Supplementary Movie 1
