# Supplementary material for: Glycaemic control boosts glucosylated nanocarrier crossing the BBB into the brain
Source: Nat Commun. 2017 Oct 17;8:1001. doi: 10.1038/s41467-017-00952-3 (PMC5645389; doi:10.1038/s41467-017-00952-3)
Supplement: Supplementary file 1 — Supplementary Information [file 41467_2017_952_MOESM1_ESM.pdf]

Supplementary Methods

Materials

α-Methoxy-ω-amino poly(ethylene glycol) (MeO-PEG-NH_2) (M_n = 2,000, M_w/M_n = 1.05) was purchased from NOF Co., Ltd. (Tokyo, Japan). β-Benzyl-L-aspartate *N*-carboxy-anhydride (BLA-NCA) was purchased from Chuo Kaseihin Co., Ltd. (Tokyo, Japan). 1,2-*O*-Isopropylidene-α-D-glucofuranoside, 1,2:5,6-di-*O*-isopropylidene-α-D-glucofuranoside (DIG), anhydrous zinc chloride, 1,5-diaminopentane (DAP) and 1-ethyl-3-(3-dimethylaminopropyl) carbodiimide hydrochloride (EDC) were purchased from Tokyo Chemical Industry Co., Ltd. (Tokyo, Japan). Ethylene oxide (EO) was obtained from Nippon Ekitan Corporation (Tokyo, Japan), and purified with CaH_2 by trap-to-trap method. Solvents used for the polymerization (THF, CH_2Cl_2 and DMF) were purified by passing through two packed columns of neutral alumina purchased from Nikko Hansen & Co., Ltd. (Osaka, Japan). Benzaldehyde, *N*-methyl-2-pyrrolidone (NMP), Dulbecco's phosphate-buffered saline (D-PBS(-)), D-(+)-glucose and 4% paraformaldehyde in phosphate buffer were purchased from Wako Pure Chemical Industries, Ltd. (Osaka, Japan). Sulfo-Cyanine 5 succinimidyl ester (Cy5-NHS) was purchased from Lumiprobe Corporation (Hallandale Beach, FL). Cell lysis buffer was purchased from Promega Corporation (Madison, WI). Anti-PECAM1 antibody (557355) was obtained from BD Biosciences (Franklin Lakes, NJ). Anti-Tuj1 antibody (MMS-435P) was purchased from Covance (Princeton, NJ). Anti-Iba1 (019-19741) was obtained from Wako Pure Chemical Industries, Ltd. Anti-GFAP (ab16997) and anti-GLUT1 (ab40084) antibodies were purchased from Abcam (Cambridge, UK). Anti-Rab11a antibody (#2413S) was obtained from Cell Signaling Technology (Danvers, MA). Alexa Fluor 488 conjugated goat anti-

rabbit IgG (A11034), goat anti-rat IgG(H+L) (A11006) and ProLong® Gold antifade reagent with DAPI were purchased from Invitrogen Molecular Probes (Eugene, OR).

Animals

BALB/c mice (female; 5-weeks-old) were purchased from Charles River Laboratories Japan, Inc. (Yokohama, Japan). All animal experiments were carried out in accordance with the guidelines at The University of Tokyo and Tokyo Medical and Dental University.

Polymer characterization

The degree of polymerization (DP) was verified by ^{1}H-NMR spectroscopy [JEOL ECS400 (JEOL Ltd., Tokyo, Japan); solvent: D_2O or d_6-DMSO]. The molecular weight distribution of polymers was determined by gel permeation chromatography [column: superdex 200 10/300 (GE Healthcare, Little Chalfont, UK); eluent: 10 mM phosphate buffer (pH 7.4) containing 500 mM NaCl for polyanion, 10 mM acetic acid aq. containing 500 mM NaCl for polycation; flow rate: 0.75 mL/min].

Synthesis of CH_3O-PEG-PAsp and CH_3O-PEG-P(Asp-AP)-Cy5 block copolymers.
CH_3O-PEG-poly(α,β-aspartic acid) (CH_3O-PEG-PAsp; M_n of PEG = 2,000, DP = 75) was synthesized according to the previously reported method[1]. CH_3O-PEG-poly[(5-aminopentyl)-α,β-aspartamide]]-Cy5 (CH_3O-PEG-P(Asp-AP)-Cy5; M_n of PEG = 2,000, DP = P(Asp) = 76) was prepared as follows: 500 mg of CH_3O-PEG-poly(β-benzyl-L-aspartate) (CH_3O-PEG-PBLA) block copolymer synthesized *via* reported method[2] was dissolved in 50 mL of DMSO and added with 25 mg of sulfo-Cy5 NHS (Lumiprobe).

The mixture was purified by dialysis in DMSO for 1 week and in water for additional 1 week. After freeze drying, 363 mg (yield: 73%) of CH_3O-PEG-PBLA-Cy5 was obtained. The CH_3O-PEG-PBLA-Cy5 was reacted with DAP in accordance with the previous report[2] to obtain CH_3O-PEG-P(Asp-AP)-Cy5.

Synthesis of 3,5-*O*-benzylidene-1,2-*O*-isopropylidene-*α*-D-glucofuranoside. 3,5-*O*-benzylidene-1,2-*O*-isopropylidene-*α*-D-glucofuranoside (BIG) was synthesized according to the literature as follows[3]: 1,2-*O*-isopopylidene-*α*-D-glucofuranoside (25 g, 114 mmol) and anhydrous zinc chloride (33.3 g, 244 mmol) were mixed in benzaldehyde (100 mL, 989 mmol) at room temperature. The mixture was stirred for 5 h, then diluted with ethyl acetate (150 mL). The resulting solution was washed with water, then dried over anhydrous sodium sulfate, filtrated, evaporated at 35 °C, and finally recrystallized from hexane at 4 °C to afford BIG (5.3 g, 15 %) as a white solid. The MS spectrum was acquired by AccuTOF ESI-TOF mass spectrometer (JEOL Ltd.). MS (m/z) for $C_{16}H_{20}O_6$ ($M + H^+$): calculated 308.13, found 308.13.

Ring opening polymerization of ethylene oxide from protected glucofuranoside. The EO polymerization was performed according to our previous work with slight modification[4]. 1,2:5,6-di-*O*-isopropylidene-*α*-D-glucofuranoside (DIG, 260 mg, 1.0 mmol) or BIG (308 mg, 1.0 mmol) was sublimed in a reaction tube under vacuum at 70 °C for DIG and 150 °C for BIG. To a THF solution of DIG or BIG, 0.3 M potassium naphthalene solution in THF (3.3 mL, 1.0 mmol) was added dropwisely, and then EO (2.3 mL, 46 mmol) was charged with stirring under Ar atmosphere. After 48 h of stirring at room temperature, 1 mL of MeOH was added, and the mixture was dropped into

excess amount of ice-cold diethyl ether with stirring to obtain a white precipitate to afford DIG-PEG-OH or BIG-PEG-OH.

Terminus amination of DIG(or BIG)-PEG-OH. The *ω*-hydroxyl group of DIG-PEG-OH and BIG-PEG-OH was converted to *ω*-amino group by the following conventional procedures: A THF solution of DIG(or BIG)-PEG-OH (2.0 g, 1 mmol) and TEA (834 μL, 6.0 mmol) was added to a THF solution of methanesulfonyl chloride (387 μL, 5.0 mmol) in ice. After 6 h of stirring at room temperature, the mixture was dropped into an excess amount of ice-cold diethyl ether under continuous stirring to obtain a white precipitate, DIG(or BIG)-PEG-OMs. After drying under vacuum, the white solid was dissolved in 25% aqueous ammonia solution (200 mL) and stirred for 2 days at room temperature. The resulting solution was evaporated, dialyzed (molecular weight cut-off size (MWCO): 1,000) against dilute ammonia solution, then de-ionized water, and finally lyophilized. To remove non-aminated PEG fraction, ion-exchange chromatography was performed using sephadex C-25 (GE healthcare, Little Chalfont, UK). After collecting aminated PEG fraction, the solution was evaporated and lyophilized to obtain pure DIG(or BIG)-PEG-NH_2 as a white powder.

Preparation of DIG(or BIG)-PEG-*block*-poly(*β*-benzyl-L-aspartate) copolymer. The block copolymerization of BLA-NCA from *ω*-amino group of DIG(or BIG)-PEG was performed as previously reported. To 4 mL of CH_2Cl_2 solution of DIG(or BIG)-PEG-NH_2 (200 mg, 0.1 mmol), the solution of BLA-NCA (1.7 g, 8.5 mmol) in mixed CH_2Cl_2/DMF (10:1, v/v) was added and stirred for 2 days at 35 °C. The mixture was dropped into an excess amount of the mixture of *n*-hexane/AcOEt (6:4, v/v), and dried under vacuum. The GPC chromatogram [column: TOSOH TSKgel Super AW4000 +

Super AW3000 + Super AW3000 (TOSOH, Tokyo. Japan); eluent: NMP containing 50 mM LiBr; flow rate: 0.25 mL/min] of the obtained DIG(or BIG)-PEG-PBLA was confirmed to be unimodal with narrow distribution.

Ester hydrolysis reaction of DIG(or BIG)-PEG-PBLA. To remove benzyl ester groups in the block copolymers, alkaline hydrolysis reaction was performed. The block copolymer (1.0 g) was dissolved in 0.5 N NaOH aqueous solution (50 mL), and stirred for 1 h. The solution was then dialyzed (MWCO: 3,500) against de-ionized water, and lyophilized to obtain DIG(or BIG)-PEG-PAsp as a white powder. The quantitative deprotection of benzyl ester was confirmed from ^{1}H-NMR spectra.

Removal of protective groups from glucose moiety. DIG(or BIG)-PEG-PAsp was dissolved in TFA/water (8:2, v/v) and stirred for 1 h at room temperature. The mixture was sequentially dialyzed against aqueous 0.01 N NaOH and de-ionized water, followed by the lyophilization to obtain Gluc(6)-PEG-PAsp or Gluc(3)-PEG-PAsp. The complete removal of protective groups was confirmed, and the DP of P(Asp) was determined to be 80 for Gluc(6)- and 85 for Gluc(3) from ^{1}H-NMR spectra (D_2O, 80 °C).

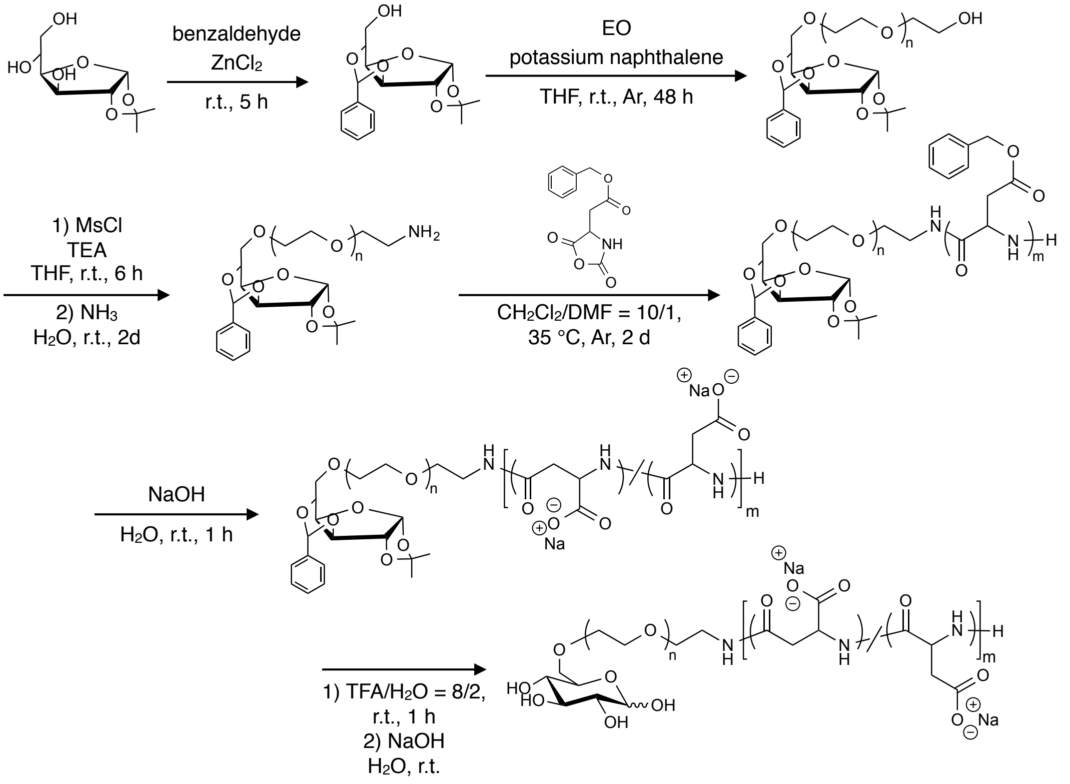

Supplementary Figure 1. Synthetic route for Gluc(6)-PEG-PAsp block copolymer.

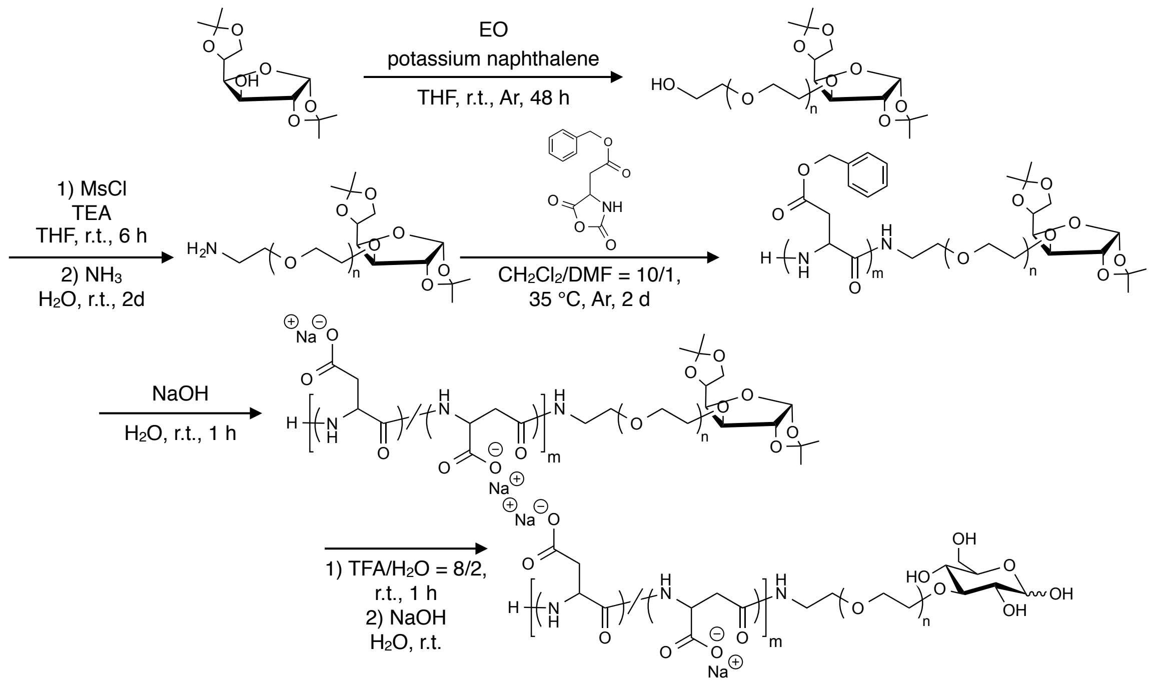

Supplementary Figure 2. Synthetic route for Gluc(3)-PEG-PAsp block copolymer.

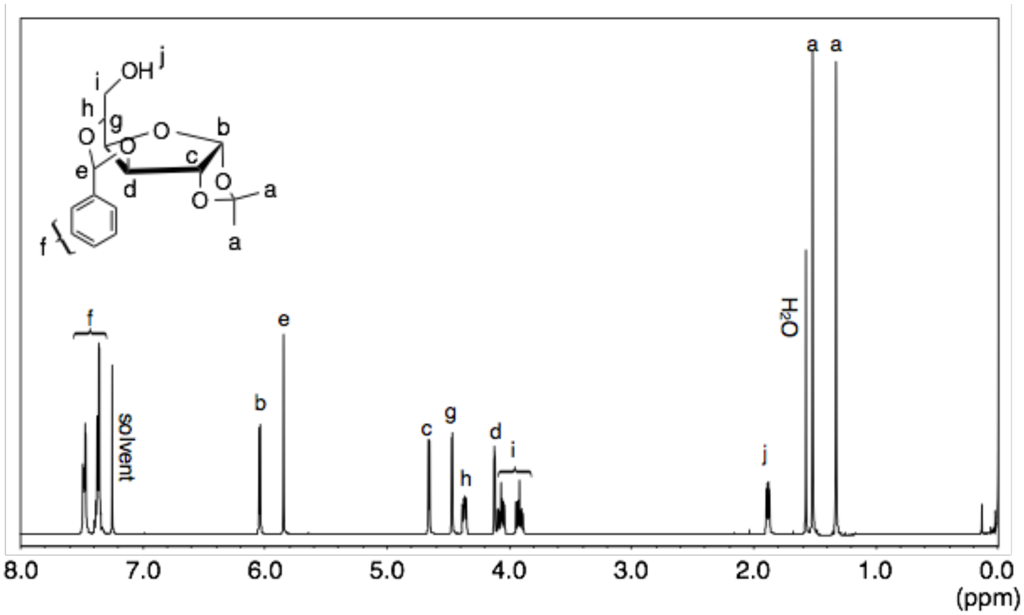

Supplementary Figure 3. ^{1}H-NMR spectrum of BIG in $CDCl_3$.

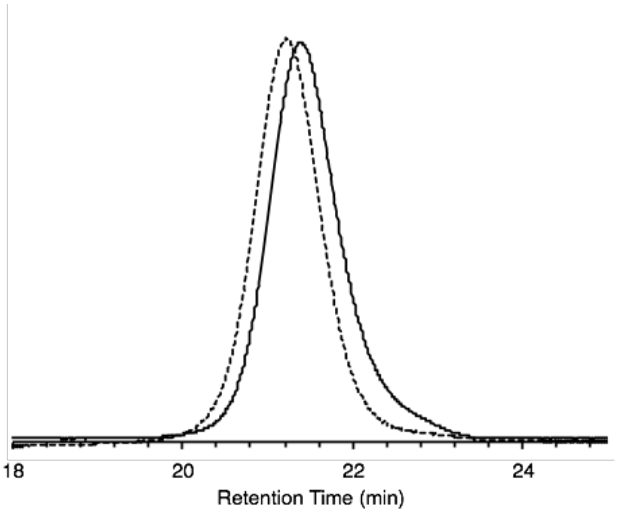

Supplementary Figure 4. GPC chromatograms of DIG(or BIG)-PEG-OH. DIG-PEG-OH (solid line) and BIG-PEG-OH (dotted line).

(A)

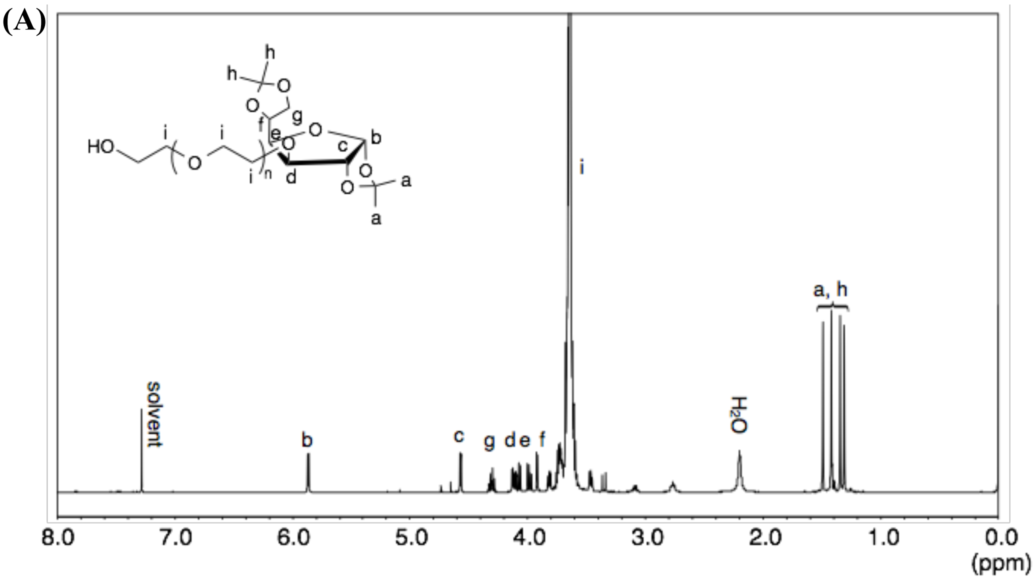

(B)

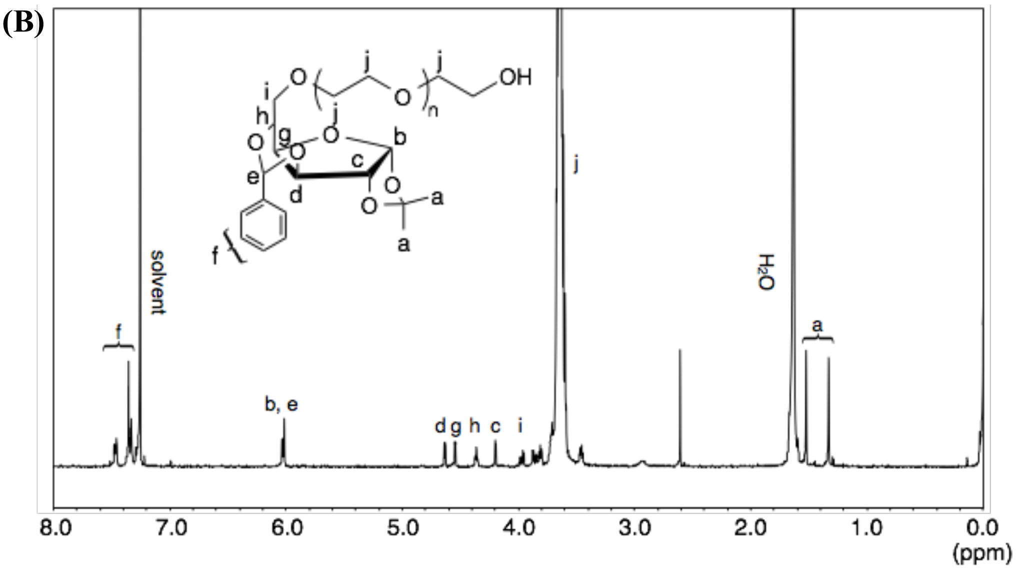

Supplementary Figure 5. ^{1}H-NMR spectra of DIG-PEG-OH (A) and BIG-PEG-OH (B) in $CDCl_3$.

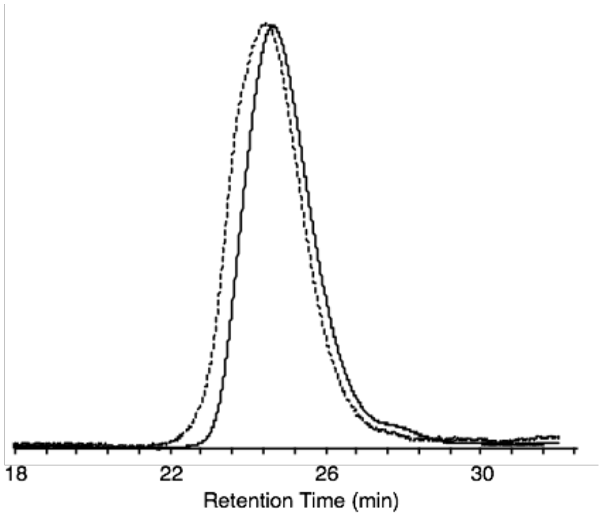

Supplementary Figure 6. GPC chromatograms of DIG-PEG-PBLA and BIG-PEG-PBLA block copolymers. DIG-PEG-PBLA (solid line) and BIG-PEG-PBLA (dotted line).

(A)

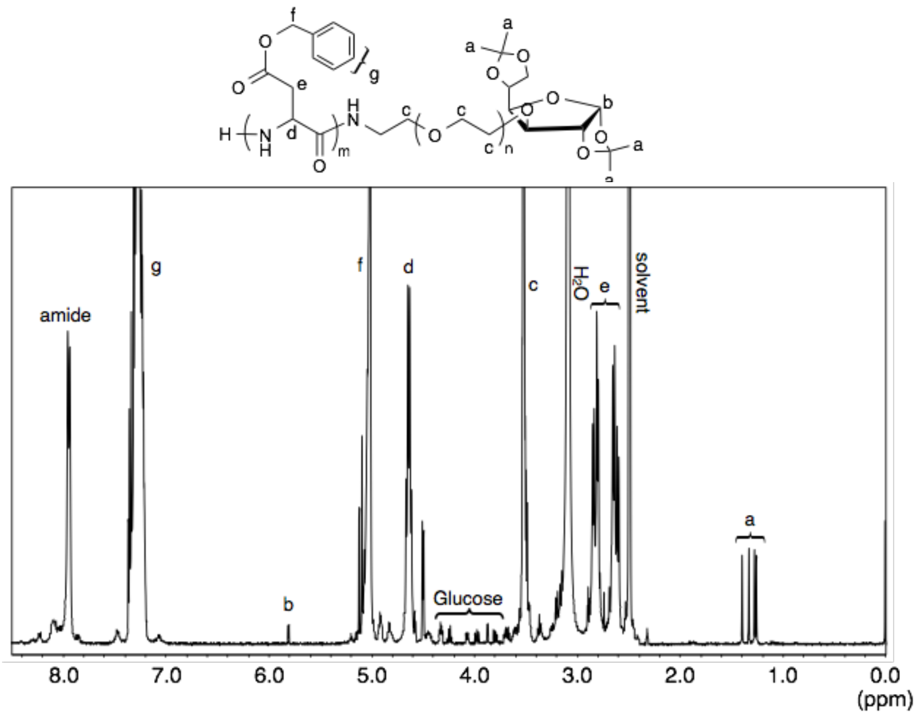

(B)

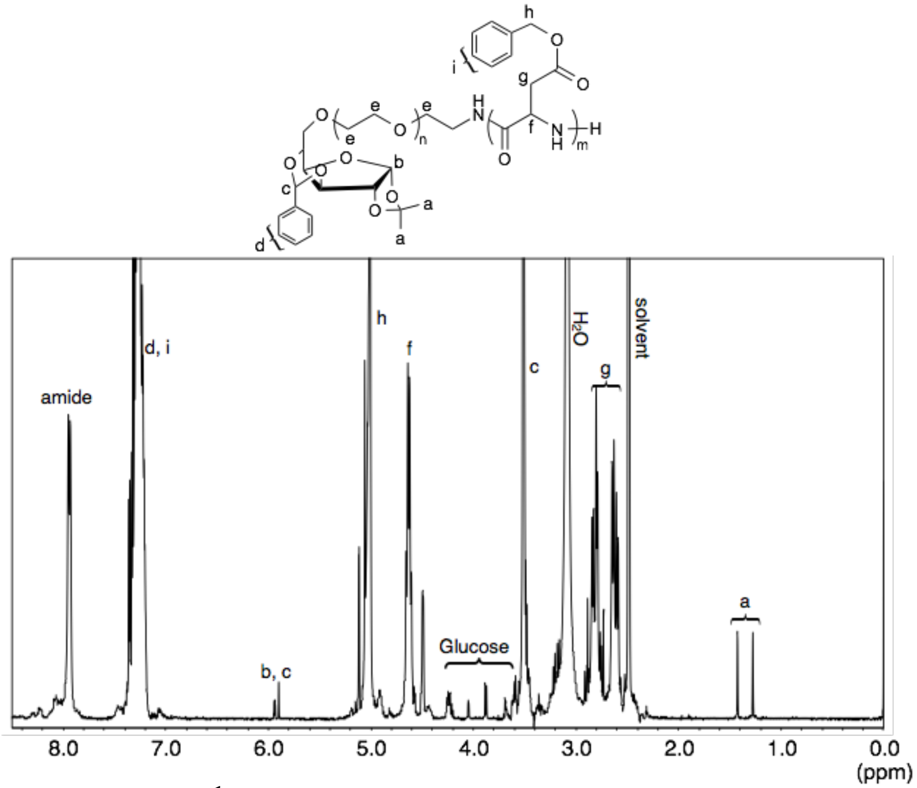

Supplementary Figure 7. ^{1}H-NMR spectra of DIG-PEG-PBLA (A) and BIG-PEG-PBLA (B) block copolymers in d_6-DMSO.

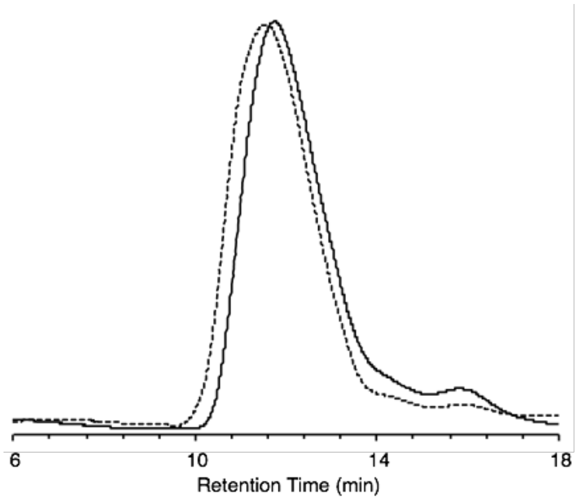

Supplementary Figure 8. GPC chromatograms of DIG-PEG-PAsp and BIG-PEG-PAsp block copolymers. DIG-PEG-PAsp (solid line) and BIG-PEG-PAsp (dotted line).

(A)

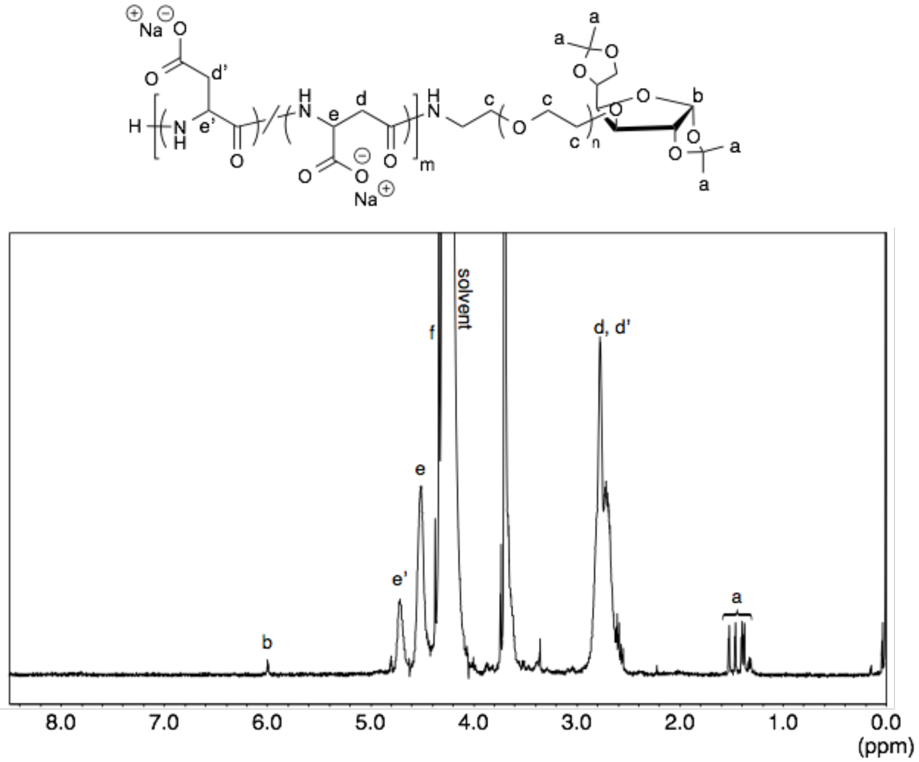

(B)

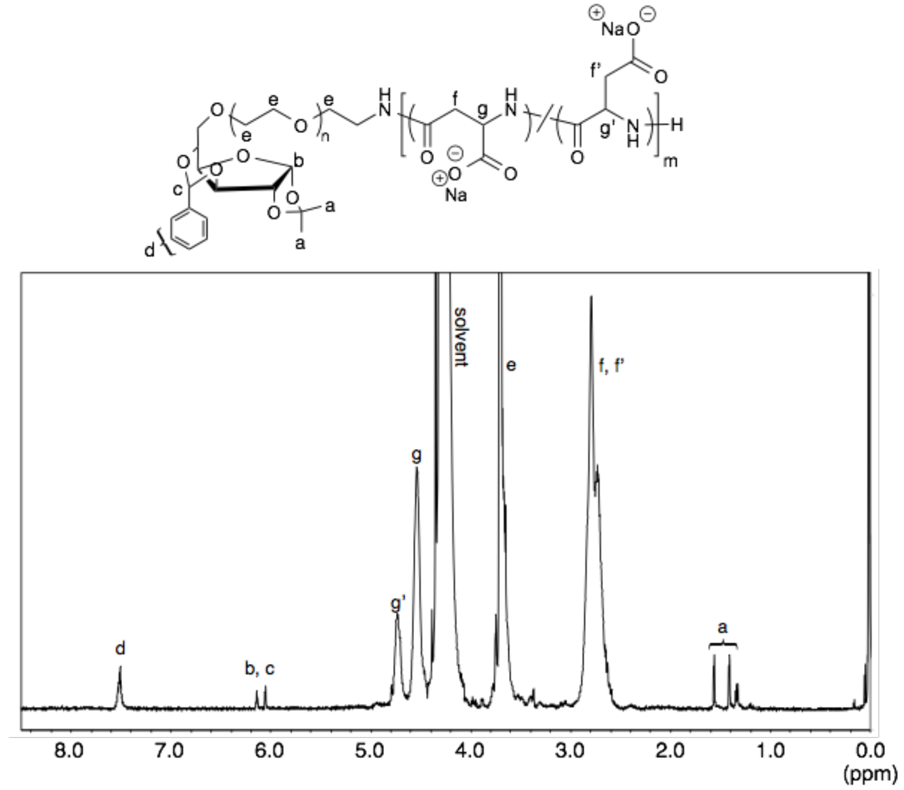

Supplementary Figure 9. ^{1}H-NMR spectra of DIG-PEG-PAsp (A) and BIG-PEG-PAsp (B) block copolymers in D_2O.

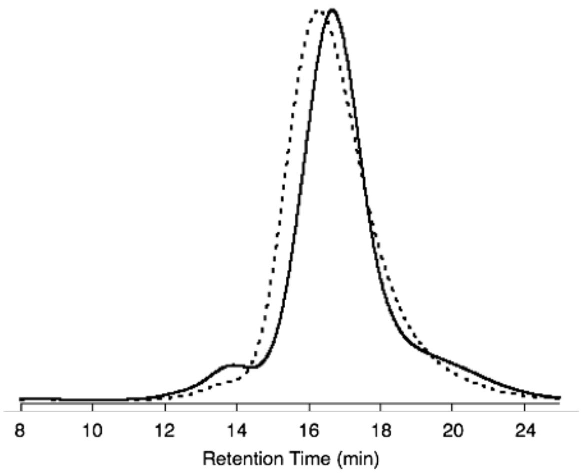

Supplementary Figure 10. GPC chromatograms of Gluc(3)-PEG-PAsp and Gluc(6)-PEG-PAsp block copolymers. Gluc(3)-PEG-PAsp (solid line) and Gluc(6)-PEG-PAsp (dotted line).

(A)

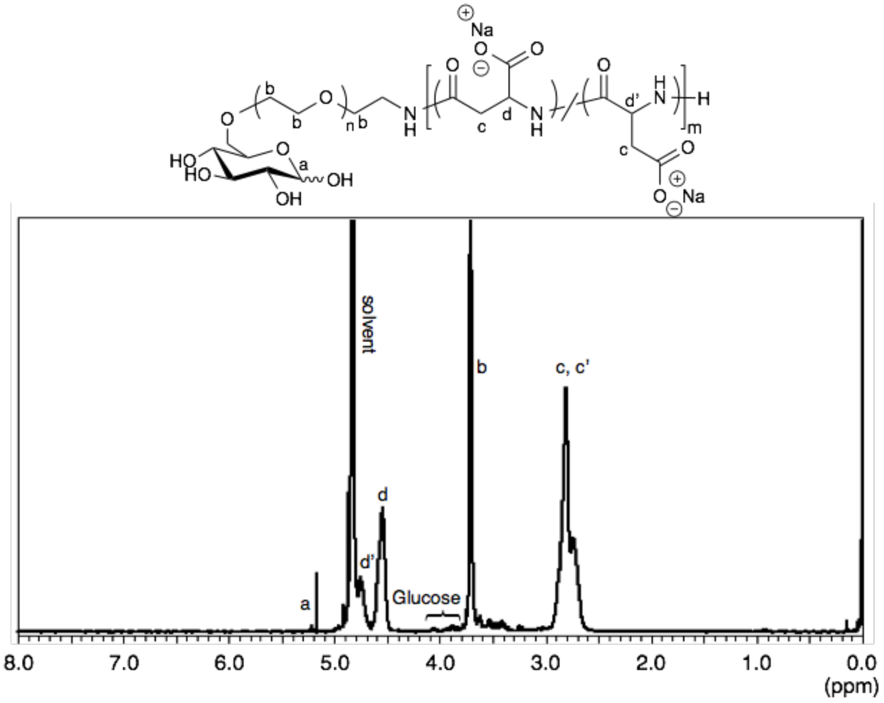

(B)

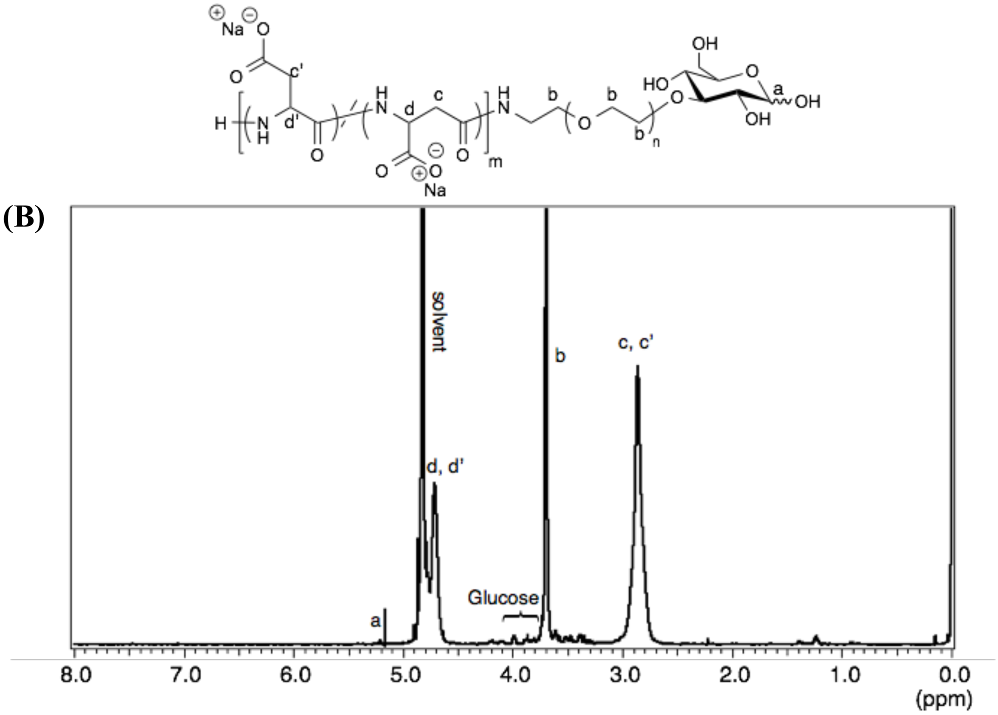

Supplementary Figure 11. ^{1}H-NMR spectra of Gluc(3)-PEG-PAsp (A) and Gluc(6)-PEG-PAsp (B) block copolymers in D_2O.

Gluc(6)/m (Gluc(3)/m, Null/m) preparation.

Solutions of block copolymers at 1 mg/mL (Gluc(6)-PEG-P(Asp), CH_3O-PEG-P(Asp), CH_3O-PEG-P(Asp-AP) and CH_3O-PEG-P(Asp-AP)-Cy5) were prepared in 10 mM phosphate buffer (pH 7.4, 0 mM NaCl) and passed through a 0.22 μm membrane filter to remove dust particulates. The CH_3O-PEG-P(Asp-AP) and CH_3O-PEG-P(Asp-AP)-Cy5 solutions were mixed at 4:1 residual molar ratio of amino groups to obtain the block catiomer solution, while Gluc(6)-PEG-P(Asp) and CH_3O-PEG-P(Asp) solutions were mixed at varying residual molar ratio (1:0, 1:1, and 1:4) of carboxylic groups to obtain a series of block aniomer solutions. Then, these block aniomer and block catiomer solutions were subjected to vortex-mixing at a stoichiometric residual molar ratio of amino groups to carboxyl groups to form Gluc(6)/m with varying fraction (10, 25, and 50%) of glucosylated PEG strands in the PEG shell layer. All of these Gluc(6)/m were then crosslinked by adding 10 mg/mL EDC in 10 mM phosphate buffer at 10 equivalent amounts to carboxyl groups, and maintained overnight at room temperature, followed by the purification *via* ultrafiltration with vivaspin 20 (Sartorius stedim Biotech GmbH, Goettingen, Germany) [MWCO: 100,000]. The preparation of Gluc(3)/m and Null/m was performed in the similar manner to the above description by changing Gluc(6)-PEG-P(Asp) to Gluc(3)-PEG-P(Asp) for Gluc(3)/m or CH_3O-PEG-P(Asp) for Null/m.

Dynamic light scattering (DLS) analysis.

The size distribution of Gluc(6)/m, Gluc(3)/m and Null/m was evaluated by DLS measurements at 25 ^{o}C in 10 mM phosphate buffer at pH 7.4 using a Zetasizer Nano ZS90 (Malvern Instruments Ltd., Worcestershire, UK).

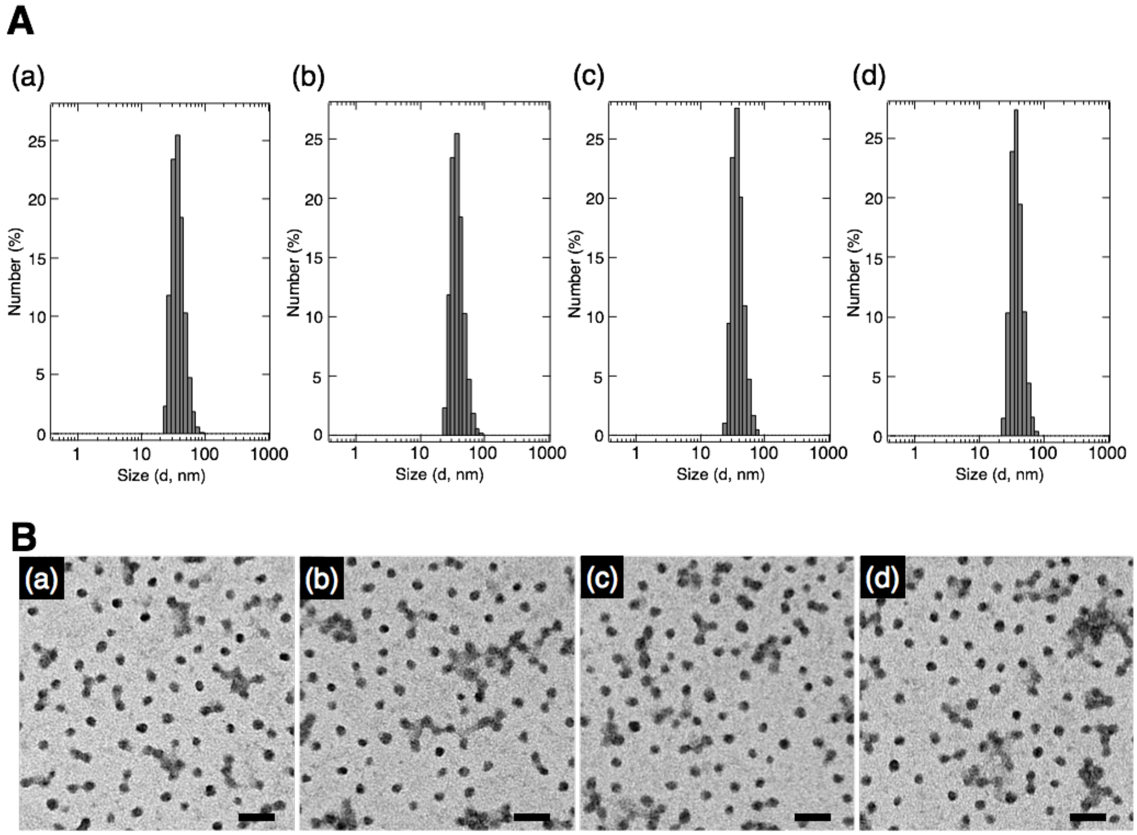

Supplementary Figure 12. Characterization of the polymeric micelles. (A) Representative size distributions of (a) Null/m, (b) 10%Gluc(6)/m, (c) 50%Gluc(6)/m and (d) 25%Gluc(3)/m determined by DLS. (B) TEM image of (a) Null/m, (b) 10%Gluc(6)/m, (c) 50%Gluc(6)/m and (d) 25%Gluc(3)/m. The scale bar indicates 100 nm.

Micelles	Size (nm)	PDI
Null/m	30	0.054
10%Gluc(6)/m	33	0.075
25%Gluc(6)/m	32	0.065
50%Gluc(6)/m	35	0.078
25%Gluc(3)/m	32	0.055

Supplementary Table 1. Size of various micelles prepared from different combination of block copolymers determined by DLS.

Generation of GLUT1-overexpressing stable cell line.

Neuro2a cells were co-transfected with either mouse GLUT1 expressing plasmid (OriGene, Rockville, MD) or Mock plasmid, and pAcGFP1 (Clontech laboratories, Mountain View, CA). In brief, cells were seeded on 10cm plate and cultivated for 18 h and then transfected with 4 µg of total DNA (mouse GLUT1 plasmid: pAcGFP1 or Mock plasmid: pAcGFP1, 10:1) by Lipofectamine 2000 (Invitrogen) according to the manufacturer's protocol. After 24 h, the culture medium was exchanged to the fresh medium containing 400 µg/ml G418 (Roche Diagnostics, Basel, Switzerland) for the selection. The stable clones expressing GFP were picked after 10 days under the fluorescent microscope and seeded into individual wells of a 24 well plate with the selection medium. The stable cell line was expanded for 7 days before the clonal populations were profiled for GLUT1 expression as follows.

Western blotting was performed to analyze GLUT1 protein levels. Cells were harvested with homogenate buffer [10 mM Tris-HCl (pH 7.4), 150 mM NaCl, 1 mM EDTA, 4% CHAPS, 1× Complete protease inhibitor cocktail (Roche Diagnostics)]. 10 µg of extracted proteins were mixed with Laemmli sample buffer (Bio-Rad, Hercules, CA), denatured at room temperature for 30 min, and separated on a 5%–20% gradient polyacrylamide gel (ATTO Corporation, Tokyo, Japan). The separated proteins were transferred to a polyvinylidene difluoride membranes (Bio-Rad). Blots were probed with primary antibodies against GLUT1 (1:500, Alpha Diagnostic, San Antonio, TX) or GAPDH (1:5000, Sigma-Aldrich, St. Louis, MO), and then incubated with anti-rabbit secondary antibody (1:2000, Santa Cruz Biotechnology, Santa Cruz, CA), anti-mouse secondary antibody (1:2000, Santa Cruz Biotechnology) conjugated with horseradish

peroxidase. Blots were visualized with SuperSignal West Femto Maximum Sensitivity Substrate (Thermo Fisher Scientific, Waltham, MA) and analyzed by a ChemiDoc Touch Imaging System (Bio-Rad).

Immunocytochemistry was also performed to check the expression of GLUT1. GLUT1-overexpressing cells or mock-transfected cells were grown in collagen-I coated 4-well culture slides. The cells were fixed in 4% paraformaldehyde for 30 min at room temperature and then stained with DAPI to visualize the nuclei and immunolabelled with anti-GLUT1 antibody (1:200, Abcam). This was followed by incubation with an Alexa Fluor 488-conjugated secondary antibody. All images were acquired with A1R confocal laser scanning microscope (Nikon Corp., Tokyo, Japan).

Inhibition assay of polymeric micelle uptake into GLUT1-overexpressing cells.

GLUT1-overexpressing cells were grown in 96-well plate and incubated at 37 ^{o}C. The cells were treated with either cytochalasin B (Sigma-Aldrich., at 20 μM or 100 μM) or phloretin (Wako Pure Chemical Industries Ltd., at 200 μM or 1 mM) for 30 min, and then Null/m, 25%Gluc(6)/m, or 50%Gluc(6)/m was added to the cell medium in which the concentration of the micelles was equivalent to that in the serum of mice injected with each micelle. After 30 min, the cells were washed by PBS twice and treated with trypsin. The amount of cell-associated micelles cells was quantified by fluorescence measurement of detached cell suspension using Infinite M1000 PRO (Tecan Group Ltd., Männedorf, Switzerland).

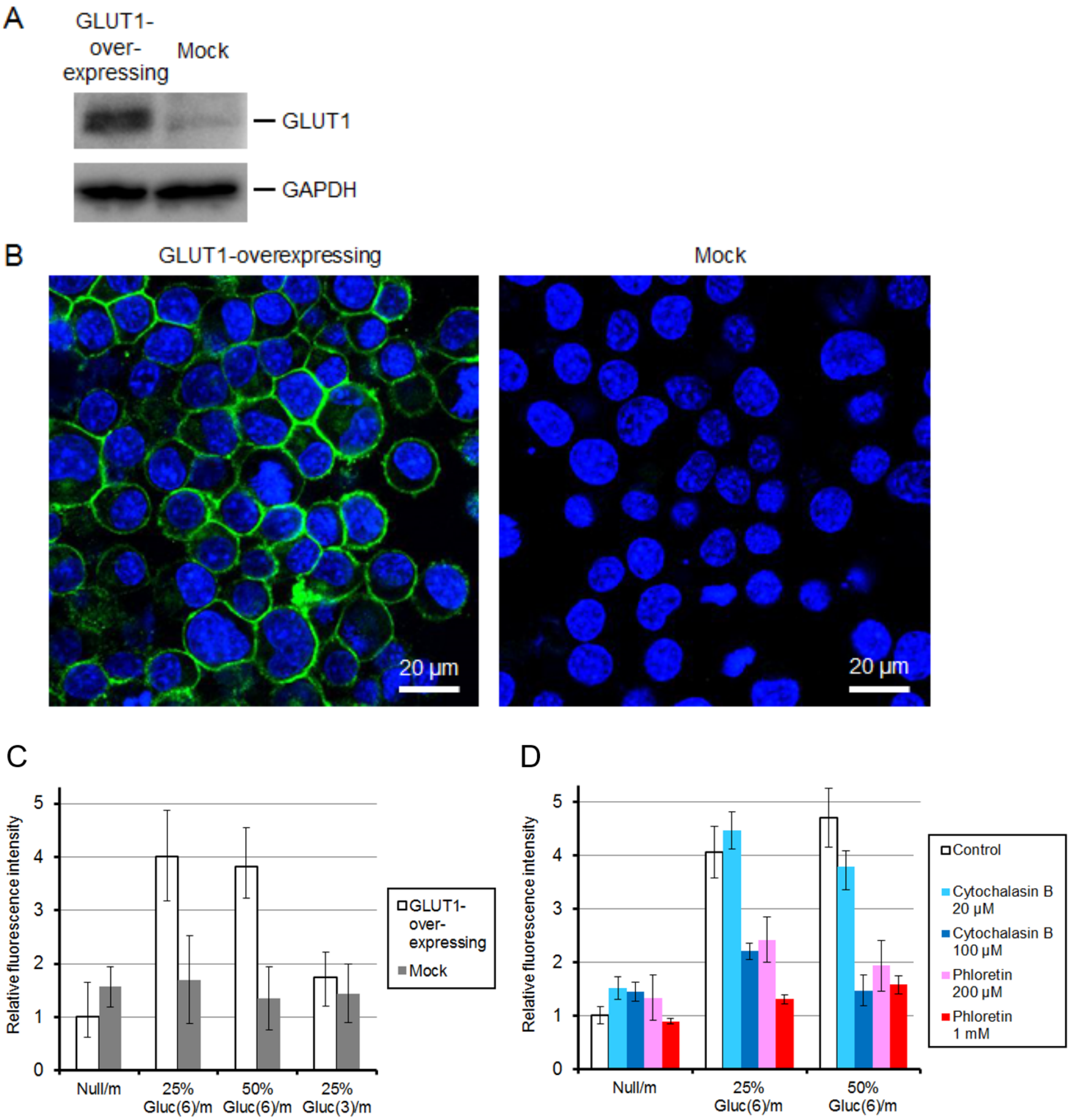

Supplementary Figure 13. Uptake of polymeric micelles into GLUT1-overexpressing cells under the varying concentration of GLUT1 inhibitors. (A) Western blotting of GLUT1 for GLUT1-overexpressing and mock-transfected cells (B) Immunocytochemistry of GLUT1 for GLUT1-overexpressing and mock-transfected cells

(C) Uptake of Null/m, 25%Gluc(6)/m, 50%Gluc(6)/m, and 25%Gluc(3)/m into GLUT1-overexpressing or mock-transfected cells (*n*=4). (D) Effect of GLUT1 inhibitors on the uptake of each micelle into GLUT1-overexpressing cells (*n*=4). The data are shown in relative values normalized to fluorescence intensities from the GLUT1-overexpressing cells treated with Null/m in the absence of inhibitors, and are expressed as the mean ± SEM.

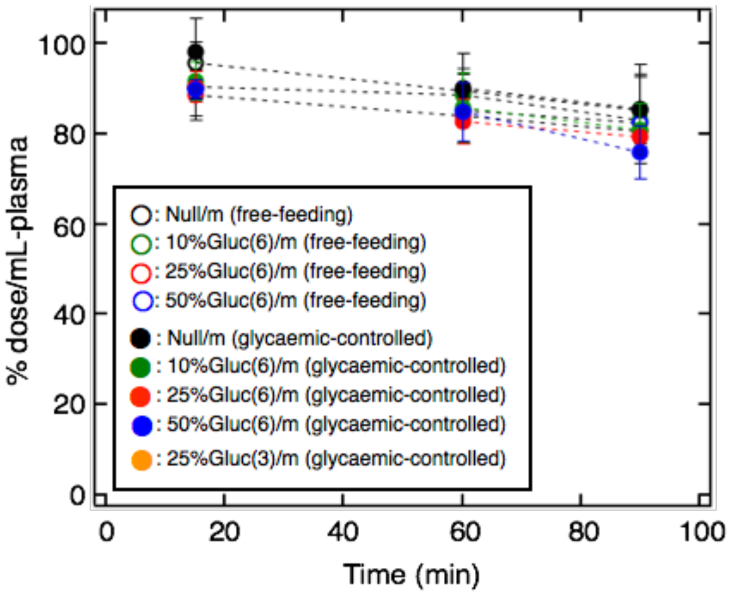

Supplementary Figure 14. Plasma retention of micelles under different feeding conditions. Open and closed circles show free-feeding and glycaemic-controlled groups, respectively. Black, green, red and blue colored circles indicate Null/m, 10%Gluc(6)/m, 25%Gluc(6)/m and 50%Gluc(6)/m, respectively. An orange colored circle shows 25%Gluc(3)/m (glycaemic-controlled). BALB/c mice (female, 6-week-old, *n*=5) were used for these experiments. The data are expressed as the mean ± SEM.

Immunohistochemical analysis of brain sections for recycling endosomes.

Mice were deprived of food for 24 h, and intravenously injected with the solution of the Cy5-labelled 25%Gluc(6)/m or 50%Gluc(6)/m, with and without an following intraperitoneal injection of 20 wt% glucose solution 30 min later. After further 30 min, the mice were sacrificed and were perfused with PBS and 4% paraformaldehyde. The brains were fixed overnight in 4% paraformaldehyde at 4 ^{o}C and sequentially soaked overnight in 20 wt% sucrose solution at 4 ^{o}C. The fixed specimens were snap-frozen in liquid nitrogen, and were then sliced into 14-μm thickness with CM3050 S cryostat (Leica Microsystems, Wetzler, Germany). The sections were stained with DAPI to visualize the nuclei and immunolabelled with antibodies against Rab11a (1:200) to visualize recycling endosomes. This was followed by incubation with an Alexa Fluor 488-conjugated secondary antibody. All images were acquired with A1R confocal laser scanning microscope (Nikon Corp.).

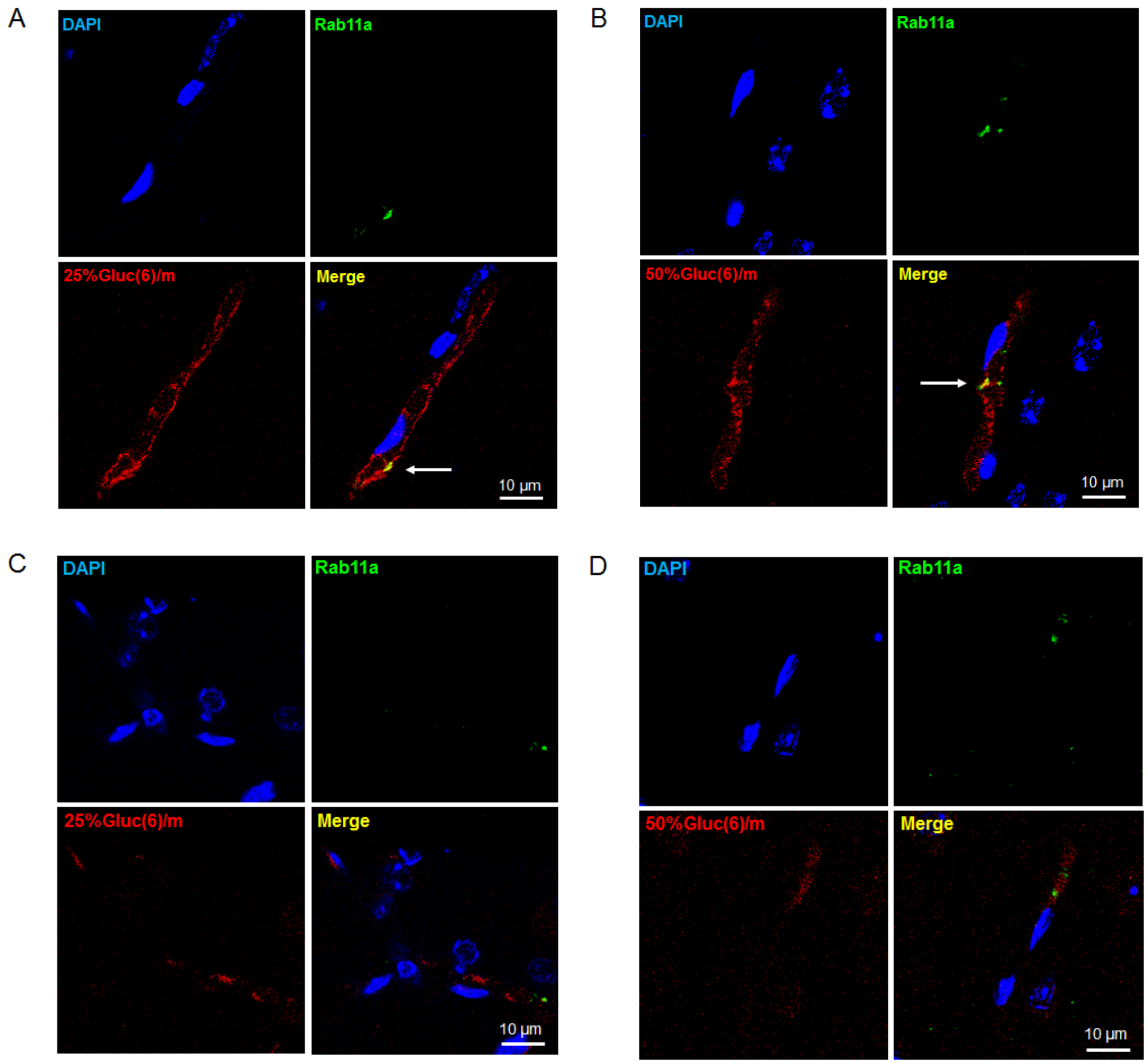

Supplementary Figure 15. Brain immunohistochemistry for recycling endosome. (A, B) Cerebral sections 60 min after an intravenous injection of 25% Gluc(6)/m (A) or 50%Gluc(6)m (B) in fasting condition with an intraperitoneal administration of 20wt% glucose solution 30 min after the injection of 25% Gluc(6)/m or 50%Gluc(6)/m. (C, D) Cerebral sections 60 min after an intravenous injection of 25% Gluc(6)/m (C) or 50%Gluc(6)m (D) in fasting condition without an intraperitoneal administration of 20wt% glucose solution. Nuclei (blue) are stained with DAPI and recycling endosomes (green) are immunolabelled with anti-Rab11a antibody.

Supplementary References

1. Anraku, Y., Kishimura, A., Oba, M., Yamasaki, Y. & Kataoka, K. Spontaneous formation of nanosized unilamellar polyion complex vesicles with tunable size and Properties. *J. Am. Chem. Soc.* **132**, 1631–1636 (2010).

2. Koide, A. *et al*. Semipermeable polymer vesicle (PICsome) self-assembled in aqueous medium from a pair of oppositely charged block copolymers: physiologically stable micro-/nanocontainers of water-soluble macromolecules. *J. Am. Chem. Soc.* **128**, 5988–5989 (2006).

3. Hung, S.-C. Methods of preparing optically pure sugars. US Patent No. 6294666 (1999).

4. Nakamura, T., Nagasaki, Y. & Kataoka, K. Synthesis of heterobifunctional poly(ethylene glycol) with a reducing monosaccharide residue at one end. *Bioconjugate Chem.* **9**, 300–303 (1998).
