# Supplementary material for: Glycaemic control boosts glucosylated nanocarrier crossing the BBB into the brain
Source: Nat Commun. 2017 Oct 17;8:1001. doi: 10.1038/s41467-017-00952-3 (PMC5645389; doi:10.1038/s41467-017-00952-3)
Supplement: Supplementary file 2 — Description of Additional Supplementary Files [file 41467_2017_952_MOESM2_ESM.pdf]

**Description of Additional Supplementary Files**

File Name: Supplementary Movie 1
Description: The movie file shows Gluc(6)/m (red) in the mouse cerebrum observed by intravital multiphoton microscopy 48 h after administration.
